# The Phytocomplex from *Fucus vesiculosus* and *Ascophyllum nodosum* Controls Postprandial Plasma Glucose Levels: An In Vitro and In Vivo Study in a Mouse Model of NASH

**DOI:** 10.3390/md15020041

**Published:** 2017-02-15

**Authors:** Daniela Gabbia, Stefano Dall’Acqua, Iole Maria Di Gangi, Sara Bogialli, Valentina Caputi, Laura Albertoni, Ilaria Marsilio, Nicola Paccagnella, Maria Carrara, Maria Cecilia Giron, Sara De Martin

**Affiliations:** 1 Department of Pharmaceutical and Pharmacological Sciences, University of Padova, 35131 Padova, Italy; daniela.gabbia@gmail.com (D.G.); stefano.dallacqua@unipd.it (S.D.A.); valekap@gmail.com (V.C.); ilaria.marsilio@gmail.com (I.M.); nicolapaccagnella89@gmail.com (N.P.); maria.carrara@unipd.it (M.C.); ceci.giron@gmail.com (M.C.G.); 2 Department of Chemical Sciences, University of Padova, 35131 Padova, Italy; iolemaria.digangi@unipd.it (I.M.D.G.); sara.bogialli@unipd.it (S.B.); 3 Department of Medicine, General Pathology and Cytopathology Unit, University of Padova, 35128 Padova, Italy; aoichan@libero.it

**Keywords:** *Ascophyllum nodosum*, *Fucus vesiculosus*, nonalcoholic steatohepatitis, postprandial blood glucose level

## Abstract

Edible seaweeds have been consumed by Asian coastal communities since ancient times. *Fucus vesiculosus* and *Ascophyllum nodosum* extracts have been traditionally used for the treatment of obesity and several gastrointestinal diseases. We evaluated the ability of extracts obtained from these algae to inhibit the digestive enzymes α-amylase and α-glucosidase in vitro, and control postprandial plasma glucose levels in a mouse model of non-alcoholic steatohepatitis (NASH); a liver disease often preceding the development of Type 2 diabetes (T2DM). This model was obtained by the administration of a high-fat diet. Our results demonstrate that these algae only delayed and reduced the peak of blood glucose (*p* < 0.05) in mice fed with normal diet, without changing the area under the blood glucose curve (AUC). In the model of NASH, the phytocomplex was able to reduce both the postprandial glycaemic peak, and the AUC. The administration of the extract in a diet particularly rich in fat is associated with a delay in carbohydrate digestion, but also with a decrease in its assimilation. In conclusion, our results indicate that this algal extract may be useful in the control of carbohydrate digestion and absorption. This effect may be therapeutically exploited to prevent the transition of NASH to T2DM.

## 1. Introduction

Edible seaweed, an easily available food source, has probably been consumed by coastal communities since the dawn of time, especially in Asia [1]. Seaweeds and their organic extracts are known to contain several bioactive polysaccharides with numerous health benefits [2,3,4,5,6,7,8]. These polysaccharides cannot be completely digested by the human digestive system and represent a source of dietary fiber, prebiotics, and other functional ingredients [9,10]. Soluble fiber can slow down the digestion and absorption of nutrients by increasing the viscosity, thereby decreasing blood sugar levels and cholesterol [10]. Consistent with these observations, it has been reported that seaweed fiber consumption is associated with a significant reduction of chronic diseases, such as diabetes, obesity, and hypertension [11,12]. Recent studies have reported that fucoidan, a sulphated polysaccharide found in many species of brown algae and seaweed, has numerous biological and pharmacological activities, such as anti-proliferative, anti-inflammatory, and antiviral effects [13,14]. Algae extracts are considered a good source of digestive enzyme inhibitors. In particular, they contain polyphenolic compounds, such as bromophenols [15,16] and phlorotannins (PHTs) [5,17], which are well known α-glucosidase inhibitors. The enzyme α-glucosidase, together with α-amylase, is a key enzyme in starch breakdown and absorption. α-amylase is secreted from the pancreas and salivary glands [4], and catalyses the cleavage of α-d-(1-4) glycosidic linkages of starch, amylose, amylopectin, glycogen, and various maltodextrins, into shorter oligosaccharides, such as maltose and glucose [18,19]. α-glucosidase, which is also located in the brush-border surface membrane of intestinal cells, activates the final step of digestive processes by catalyzing the hydrolysis of complex carbohydrates and disaccharides to absorbable monosaccharides, e.g., the release of glucose from maltose and/or sucrose [20]. It has been reported that the inhibition of α-amylase and α-glucosidase can significantly lower the increase of the blood glucose level, after a mixed carbohydrate meal, by delaying the absorption of glucose [21]. In Type 2 Diabetes (T2DM) patients, this approach proves to be more efficient than controlling insulin secretion, since it is economic, convenient, and virtually prevents adverse drug reactions [22].

Nonalcoholic fatty liver disease (NAFLD) and its complication, nonalcoholic steatohepatitis (NASH), have become the most common causes of chronic liver disease in Western countries, causing considerable liver-related morbidity and mortality [23]. Evidence has accumulated that NAFLD may precede the development of T2DM [24], since one hallmark of this liver condition is insulin resistance, which is strongly associated with T2DM and abdominal obesity [24]. The subset of NAFLD patients who have NASH is estimated to be about 15% of the European [25] and USA [26] populations. Patients with NASH are at great risk of developing progressive liver disease and associated morbidity. Interestingly, the prevalence of NASH significantly increases in high-risk populations, since NASH is present in 25%–30% of patients with obesity or T2DM, and more than 35% of severely obese patients who have T2DM [27,28,29].

In this study, we analyzed in vitro inhibitory activity on the digestive enzymes α-amylase and α-glucosidase of, a commercially-available extract of *Ascophyllum nodosum* (egg wrack), and *Fucus vesiculosus* (bladder wrack). Following this, we studied its effect on blood glucose levels after starch ingestion in a mouse model of NASH, obtained by the administration of a high-fat diet (HFD) for five weeks [30,31]. Since NASH is often associated with insulin resistance and T2DM, the objective of this study was to ascertain whether seaweed extracts may be useful for glycaemic control in this liver condition.

## 2. Materials and Methods

### 2.1. Chemicals and Reagents

Algae extract, commercially available under the trade name Gdue™ (Lot. N. 201400566), was provided by Aesculapius (Brescia, Italy). The extract was prepared from the dried thallus of *Ascophyllum nodosum* and *Fucus vesiculosus*, using a proprietary hot-water extraction process, followed by a series of filtration and ultrafiltration processes, and completed by spray-drying. Alginates and part of salts were removed during the process. The extract constituted a whole plant extract that contained plant polyphenols (35.5%, as indicated in the certificate of analysis of the commercial product), in addition to the algal polysaccharides (fibers) and minerals (iodine content <300 mg/kg).

Starch potato, dipotassium hydrogen phosphate (K_2_HPO_4_), potassium dihydrogen phosphate (KH_2_PO_4_), sodium potassium tartrate, sodium hydroxide (NaOH), sodium chloride (NaCl), glutathione, human salivary α-amylase, *α*-glucosidase, acarbose, p-nitrophenyl-*α*-d-glucopyranoside (PNP-Gluc), and dinitrosalicylic acid (DNS), were obtained from Sigma Aldrich (St. Louis, MO, USA).

### 2.2. NMR, HPLC-DAD, and GC-MS Analysis

For fingerprinting of the extract, ^1^H-NMR spectra were acquired using a Bruker Avance III spectrometer (Billerica, MA, USA), operating at 400 MHz. The samples (100 mg) were weighted and extracted with 1 mL of solvent (deuterated methanol or deuterated water) in an ultrasonic bath, for 10 min. Samples were centrifuged (13,000 rpm) and the supernatant was transferred to an NMR tube to be measured.

For the analysis of the polysaccharides, a Tosohaas PWXL 5000 9.4 × 300 mm was used as a stationary phase. An Evaporative Light Scattering Detector (Agilent, Santa Clara, CA, USA) was used to detect polysaccharides and a diode array detector was employed to observe absorption at 280 nm and the UV spectra. Elution was obtained using a mixture of 10 mM water and ammonium formiate with 1% of acetonitrile. The flow rate was set at 1 mL/min. Different dextrans (1000, 5000, 80,000, 150,000 Da) were used to create a calibration between the molecular weight and retention times. An estimation of the molecular weight of the polysaccharide fraction was obtained by comparing the retention times with the standard dextrans.

GC-MS analysis of fatty acids was performed using a TRACE DSQ instrument (Thermo Scientific, Waltham, MA, USA), with a chromatographic column DB5 (30 m × 0.25 mm × 0.25 pm) and a helium gas flow rate of 1.2 mL/min. Chromatographic separation was performed using the following program: 100 °C for 4 min, an increase of 10 °C/min up to 200 °C, followed by an increase of 4 °C/min up to 260 °C. The algal sample was derivatized in order to detect the presence of the methyl esters of fatty acids, and to compare this with methylated standards.

### 2.3. α-Amylase Activity Assay

A chromogenic 3,5-dinitrosalicylic acid (DNS) assay was employed to assess α-amylase activity, as described by Roy et al. [32]. Potato starch was solubilized by boiling a 1% solution for 15 min in 20 mM sodium phosphate (pH 6.9), containing 6.7 mM NaCl. The assay was carried out at 20 °C for 5 min in a final volume of 2.1 mL with α-amylase (0.83 μg/mL) and soluble starch (47.6 μg/mL). The inhibitory effect was measured by incubating 1 mL of substrate with 100 μL of algae extract solution at 20 °C for 10 min, prior to the assay. The reaction was stopped by adding 1 mL of DNS reagent and by boiling for 15 min. After the addition of 9 mL of water, the absorbance was read at 540 nm. The inhibitory activity was calculated by means of the following formula:

$$\%\;Inhibition=\frac{A_{540Control}-A_{540Test}}{A_{540Control}}\times 100$$

### 2.4. α-Glucosidase Activity Assay

α-Glucosidase activity was measured as the amount of p-nitrophenol released from PNP-Gluc [33]. The reaction was carried out at 37 °C for 20 min in 57 mM sodium phosphate buffer (pH 6.8 °C), containing 0.1 mM glutathione, 0.85 mM p-nitrophenylpyranoside (PNP)-Gluc, and 0.2 U α-glucosidase. The reaction was stopped by adding 100 mM of sodium carbonate. The inhibitory effect was measured by performing the reaction in the presence of 100 μL of algae extract or 100 μL of water (control). The production of p-nitrophenol was quantified by measuring the absorbance at 400 nm, and the inhibitory percentage was calculated by means of the following formula:

$$\%\;Inhibition=\frac{A_{400Control}-A_{400Test}}{A_{400Control}}\times 100$$

### 2.5. Animals and Treatments

All experimental protocols were performed after authorization from the Animal Care and Use Ethics Committee of the University of Padova and the Italian Ministry of Health (no. 80/2011-B, 29 April 2011), and were in compliance with national and European guidelines for the handling and use of experimental animals.

This investigation was performed on 60 C57BL/6J mice (8 ± 2 weeks old; Charles River Laboratories, Calco (Lecco), Italy), which were randomly assigned to two experimental groups: one fed with either standard, and the other with a high-fat diet (60/Fat, kcal from: 23.5% protein, 18.4% carbohydrate; 60.3% fat; Harlan Laboratories, Lesmo (Monza Brianza) Italy) for 35 days. Mice had access to their diet and water ad libitum, were housed at 22 °C under a 12 h light/dark cycle, and were kept as four specimens per cage, in the same room, by the same personnel. The body weight was measured once a week. At day 35, all mice were fed by gavage with a 50%–50% starch (Sigma Aldrich, St. Louis, MO, USA) and safflower oil solution (2 mL/kg·bw). Each group (standard and high-fat diet) was divided into two subgroups: one served as a control and the other was treated with algae extract, which was dissolved in the starch solution to reach a concentration of 7.5 mg/kg·bw. Blood glucose levels were measured 30, 60, 120, and 180 min after gavage. Insulin levels were measured 0, 30, 60, and 180 min after gavage, by means of an ELISA kit (EMD Millipore, Darmstadt, Germany), following the manufacturer’s instructions.

### 2.6. Histological Analysis

In order to evaluate the presence of fibrosis and/or steatosis, histological analyses were performed on liver tissue samples fixed in 4% formaldehyde and embedded in paraffin. Five-micrometer sections were stained with hematoxylin-eosin and picric acid-acid fuchsin (Van Gieson stain), using standard techniques. Histological examinations of liver tissue were all performed by the same observer, who was blinded from any information about the mouse. Images were obtained by means of a Leica DM LB2 microscope equipped with a Leica DFC280 camera (Wetzlar, Germany).

### 2.7. Statistical Analysis

Statistical analyses were performed by means of the GraphPad Prism software, version 5 (GraphPad Software Inc., San Diego, CA, USA). Unless otherwise stated, the data are presented as mean values ± S.E.M. A comparison of the experimental data obtained from the different groups was made by one-way analysis of variance (ANOVA), followed by the Newman-Keuls *post-hoc* test, or by the Student’s *t*-test, when appropriate. A comparison of the results obtained from the control specimens and the animals treated with algae extract was performed by means of a Student’s *t*-test. *p* < 0.05 was considered statistically significant.

## 3. Results

### 3.1. Phytochemical Fingerprint of the Algal Extract

As a first step for the profiling of the constituents present in the algal extract, ^1^H-NMR was used. Two samples were prepared using deuterated methanol (MeOD) and deuterated water (D_2_O). The ^1^H-NMR of the extract in MeOD (Figure S1) showed the presence of signals in the range of δ 0.85–5.00 ppm. No signals were visible in the field region ascribable to the aromatic protons, thus supporting the argument that poor amounts of aromatic compounds bearing hydrogen atoms are present, or that such constituents are not soluble in methanol. Further signals in the aliphatic region support the presence of fatty acid derivatives, due to a broad triplet at δ 0.89 ppm which is ascribable to the terminal methyl group, broad multiplets at δ 1.29–1.33 ppm typical of aliphatic CH_2_, and triplets at δ 2.03 and 2.35 ppm which are ascribable to the CH_2_ nearby double bond and carbonyl function, respectively. Further signals at δ 5.14 and 5.35 ppm can support the presence of double bonds in the fatty acid chain. A singlet at δ 2.16 ppm can be assigned to the acetyl methyl group. Other signals in the spectrum can also reveal the presence of sugar residues, namely the multiplets at δ 3.50–3.80 ppm and the doublet of doublets at δ 4.16–4.35 ppm, which can be ascribed to CH_2_ of glycerol. Signals ascribable to ethanol presence are also visible as quartet at δ 2.33 ppm and triplet at δ 1.18 ppm, presented as a relative amount of 2:3.

The ^1^H-NMR dissolved in D_2_O only showed signals in the range of δ 3.50–3.90 ppm, being ascribable to oligosaccharide or sugar portions (Figure S2). Two small singlets at δ 8.40 and 6.45 ppm are visible in the spectrum after water suppression with presaturation. Additionally, broad signals in the range δ 6.00–6.35 ppm are visible and can be assigned to phenolic protons. A previous study indicates that the signals in the ^1^H-NMR of phlorotannins are singlets in the range of δ 6.00–6.50 ppm [34]. In the aliphatic region, minor signals are visible, namely a broad unresolved peak at 1.25 ppm, two singlets at 1.88 and 2.18 ppm, and some multiplets at δ 2.00–2.10 ppm. The number of these signals compared to the sugar region is poor, as is clearly visible in Figure S2. Thus, the NMR analysis allowed us to observe that the extract is mainly composed by saccharide derivatives and contains phloroglucinol derivatives.

HPLC with gel permeation revealed the presence of a large distribution of molecular weights for carbohydrate derivatives (Figure S3), showing that there is a large abundance of small saccharides (mono- and disaccharide), as well as large polysaccharides, in an estimated molecular weight range of 50,000 to 150,000 Da. Moreover, there are further saccharides with molecular weights larger than 150,000 Da.

The presence of a high number of fatty acids in the extract, which has been suggested by ^1^H-NMR, was confirmed by GC-MS analysis (Table 1). A typical chromatogram of this analysis is reported in Figure S4.

### 3.2. In Vitro Studies

**Figure 1:**
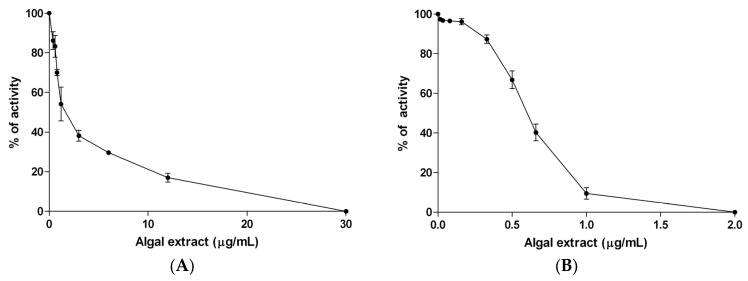
Dose-dependent inhibition of the activity α-amylase (**A**) and α-glucosidase (**B**) by the algal extract. Data are presented as means ± SD (*n* = 6). Results are obtained from three independent experiments, performed in duplicate.

The ability of algae components to inhibit α-amylase and α-glucosidase was tested by incubating these enzymes with an increasing concentration of algae extract (0.04–30 μg/mL, *n* = 8 and 0.016–2 μg/mL, *n* = 9, respectively). Our results essentially confirmed the previous observation of Roy et al. [32], that algae extract inhibits the in vitro activity of the two enzymes in a dose-dependent manner. In particular, Figure 1 shows that the complete inhibition of α-amylase and α-glucosidase activities can be reached at an algae extract concentration of 30 μg/mL and 2 μg/mL, respectively. The IC_50_ values are reported in Table 2, compared to that of the standard inhibitor acarbose. As reported in Table 2, the inhibitory potencies of algal mixtures on the two digestive enzymes are higher than those of acarbose.

### 3.3. Induction of NASH

**Figure 2:**
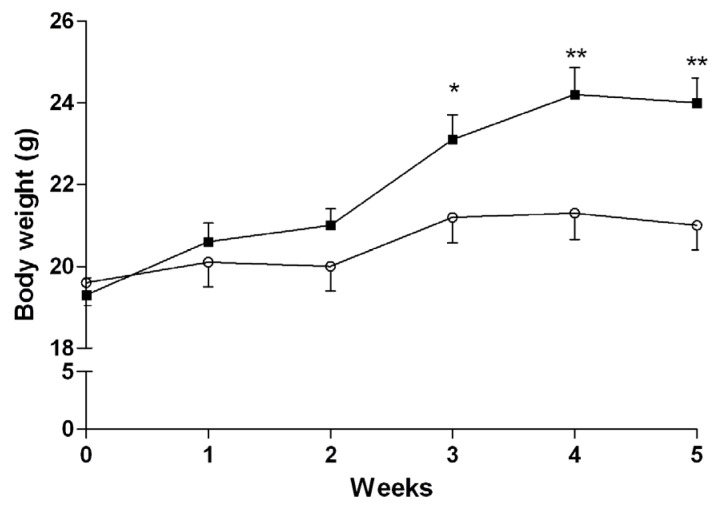
Increase of body weight of mice treated with standard (○, *n* = 30) and high-fat (■, *n* = 30) diet. Data are presented as mean ± SD. * *p* < 0.05 and ** *p* < 0.01 vs. mice treated with standard diet, Student’s *t*-test for unpaired data.

The effect of algae extract on plasma glucose levels was tested in vivo, in mice fed with either a normal or high-fat diet (HFD mice). As clearly shown in Figure 2, the weight of HFD mice started to be significantly higher than that of mice fed with a standard diet after three weeks of diet administration (*p* < 0.05), and remained significantly higher until the day of sacrifice (*p* < 0.01).

**Figure 3:**
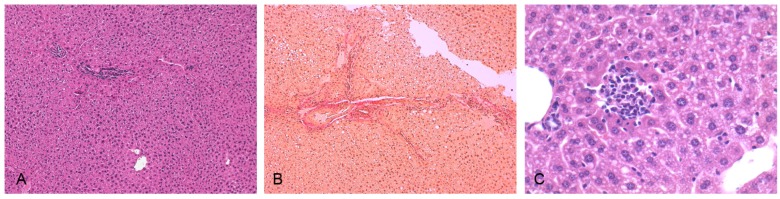
Representative photomicrographs of liver sections taken from a mouse treated with a standard diet (hematoxylin-eosin staining, (**A**) magnification 10×), and a mouse treated with a high-fat diet, stained with picric acid-acid fuchsin to detect liver fibrosis ((**B**) magnification 10×) or hematoxylin-eosin to detect liver steatosis and flogosis ((**C**) magnification 40×).

Figure 3 shows that the mice fed a normal diet showed a normal liver histology, while the mice fed with HFD showed steatosis, lobular inflammation, and periportal fibrosis (Ishak score = 1–2), proving the presence of NASH. A significant increase in the deposition of abdominal fat after the administration of HFD for five weeks (3.12% ± 0.81% vs. 1.18 ± 0.41% mice treated with HFD vs. controls; *p* < 0.01) was observed. Glycated hemoglobin, which was measured in the animals of four groups, was not significantly different (Data not shown).

### 3.4. In Vivo Studies

#### 3.4.1. Mice Fed with a Normal Diet

**Figure 4:**
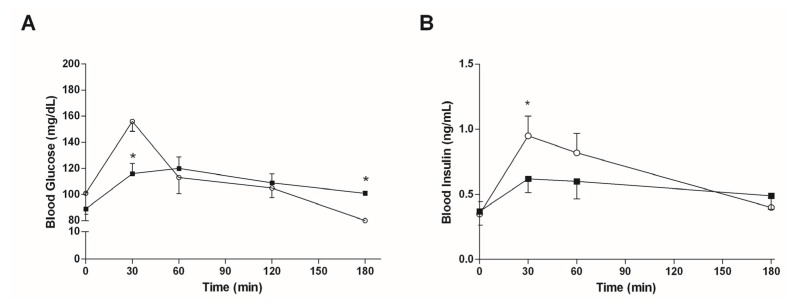
Postprandial plasma glucose (**A**) and insulin (**B**) levels in mice fed with standard diet treated with vehicle (○, *n* = 15) or 7.5 mg/kg bw of algal extract (■, *n* = 15). Data are presented as mean ± SD.* *p* < 0.05 vs. mice treated with vehicle, Student’s *t*-test for unpaired data.

Figure 4A shows that an oral intake of 7.5 mg/kg bw of algae extract reduced the initial increase of the blood sugar level which is usually observed after a starch gavage (after 30 min, 197 ± 26 mg/dL vs. 156 ± 27 mg/dL in controls and mice treated with algae extract, respectively; *p* < 0.05). There were no differences in the blood glucose levels between the two groups of mice at 60 and 120 min, whereas mice treated with the algae extract had significantly higher blood glucose levels (*p* < 0.05) 180 min after gavage. The mean AUC_0–180_ values were virtually identical (19,785 vs. 19,980 mg·min/dL in mice treated with algae extract and in controls, respectively).

The plasma insulin curves are reported in Figure 4B. The peak value for insulin is reached 30 min after gavage, and is significantly decreased by the administration of the algal extract. Accordingly, a reduction was observed in the relative AUC_0–180_ value for the same mice (98 vs. 119 ng·min/mL in mice treated with algae extract and in controls, respectively).

#### 3.4.2. Mice Fed with HFD 

**Figure 5:**
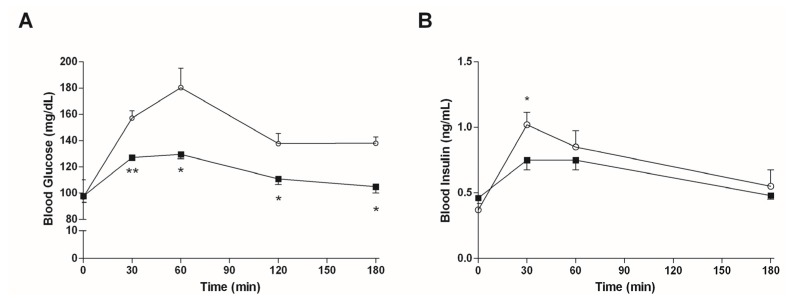
Postprandial plasma glucose (**A**) and insulin (**B**) levels in mice fed with high-fat diet treated with vehicle (○, *n* = 15) or 7.5 mg/kg bw of algal extract (■, *n* = 15). Data are presented as mean ± SD. * *p* < 0.05 and ** *p* < 0.01 vs. mice treated with vehicle, Student’s *t*-test for unpaired data.

Figure 5A shows that an oral intake of 7.5 mg/kg bw of algae extract significantly reduced the increase in the blood sugar level which is normally seen after a starch gavage in mice fed with HFD. The blood glucose levels were significantly lower in the mice treated with algae extract, with respect to the controls, at every time point of the analysis. Accordingly, the mean AUC_0–180_ value in mice treated with algae extract was found to be lower than that of controls (20,906 vs. 26,689 mg·min/dL, respectively).

As shown in Figure 5B, in NASH mice, the insulin peak is reached 30 min after gavage and is significantly decreased by the administration of the algal extract. AUC values confirm a reduction in insulin secretion (114 vs. 132 ng·min/mL in mice treated with algae extract and in controls, respectively).

## 4. Discussion

Preclinical and clinical studies have already demonstrated that algal extracts can reduce glucose release from maltose and/or sucrose by inhibiting α-glucosidase [32,35]; an enzyme located in the brush-border membrane of the small intestine. α-Glucosidase inhibitors, such as acarbose and voglibose [36,37], are widely used for the treatment of T2D.

In this study, we investigated the in vitro inhibitory effect of a phytocomplex extracted from *Fucus vesiculosus* and *Ascophyllum nodosum*, on both α-glucosidase and α-amylase; two intestinal enzymes involved in carbohydrate digestion. The ability of a phytocomplex obtained from these algae to inhibit both enzymes has already been demonstrated by Roy et al. [32], and was confirmed in this research. In particular, the IC_50_ value of this extract for α-amylase inhibition was 10 to 1000-fold lower than that reported for algae extracts prepared with solvents [17], fruit extracts [38], or polyphenols obtained from other plants [39,40]. Moreover, the Ki value (6.0 × 10^−8^ M) indicated a strong affinity of binding the algae inhibitory component to α-amylase. In this work, we confirmed these observations by demonstrating that the inhibitory potencies of algal mixtures on the two digestive enzymes are higher than that of acarbose; a prototypical α-glucosidase inhibitor. It has already been reported that this inhibitory effect is due to the high content of different bioactive compounds in the algal extract, in particular, phlorotannins (PHTs) [41,42] and fatty acids [43]. In order to ascertain the presence of these algal components, we performed a fingerprint analysis of this extract, by which we confirmed the presence of three main types of constituents: polysaccharides, polyphenolics, and fatty acids.

In order to ascertain the ability of this phytocomplex to reduce postprandial plasma glucose levels, due to the inhibition of the two intestinal enzymes, we performed an in vivo study using mice fed with either a normal or high-fat diet. The administration of HFD caused the accumulation of fat in rat livers, which, together with the deposition of fibrotic tissue, is a prototypical manifestation of NASH [23]. The modulation of the postprandial glucose level was found to be different in the two groups of mice, since the algae extract only delayed the appearance of the peak blood glucose level in mice fed with a normal diet (30 min vs. 60 min after meal in controls and mice treated with algae, respectively), without changing the area under the blood glucose curve. The blood glucose curves of mice treated with algae extract showed characteristics which are usually associated with low glycaemic index (GI) foods, i.e., a prolonged glucose absorption, since the blood glucose level of treated mice was significantly higher after 180 min with respect to controls (*p* < 0.05). This finding suggests that algal phytocomplexes can be used as dietary supplements, which, when taken before a meal, are able to slow down the rate of carbohydrate digestion and assimilation, thereby changing the glycaemic response to high GI foods into one typical of lower GI foods. In HFD mice, we demonstrated that this phytocomplex is able to affect both the postprandial glycaemic peak, which was not delayed but considerably reduced (*p* < 0.05), and the overall blood glucose level, which was lower at all measured time points, resulting in a significantly lower AUC (*p* < 0.05). Therefore, the administration of this phytocomplex in a diet particularly rich in fat is associated with a delay in carbohydrate digestion, but also with a decrease in its assimilation. Since the insulin peaks decreased significantly (Figure 4B and Figure 5B) in both healthy and NASH mice after the administration of the algal mixture, we can conclude that the decrease in plasma glucose levels is not due to an increase in insulin secretion, but more likely to a decrease of glucose absorption. The effect of the chronic administration of this algal extract, and more details about the mechanism(s) by which it affects postprandial plasma-glucose and insulin levels, and liver damage in NASH mice, is currently being investigated in our laboratory.

The results obtained from animals also support the beneficial effect of extract obtained from *A. nodosum* and *F. vesiculosus* in the modulation of postprandial blood glucose after a meal. This effect cannot be explained solely by the presence of dietary fibers in the extract, since they are known to only reduce the postprandial glycemic response when administered at higher doses (2.5 to 7.5 g in humans) [44,45]. With the current administration, the amount of dietary fibers given to animals was about 6 mg/kg·bw, which is equivalent to 420 mg in a 70-kg human being. Moreover, previous studies investigating the effect of dietary fibers in mice have reported that glucose levels were significantly lowered by the administration of at least 10% fiber in the diet [46]. These observations indicate that the observed inhibitory activity is most likely due to the phytocomplex obtained from *A. nodosum* and *F. vesiculosus*, and not to its dietary fiber content, since the amount of fiber administered in this study was lower than 10% of the food intake. Apart from phlorotannins, which are known inhibitors of digestive enzymes, as already stated in the Introduction, we identified, by means of ^1^H-NMR and GC-MS, the presence of fatty acids in the algal extract. As recently demonstrated by Bingrui and collaborators [43], the fatty acids of an algal extract can inhibit α-glucosidase [43]. Therefore, the presence of phlorotannins and fatty acids, which are both digestive enzyme inhibitors with different chemical and pharmacological characteristics, increases their pharmacological potency, as demonstrated by the fact that the IC50 values of the algal extracts which we calculated are lower than those previously reported in the literature for single components and acarbose [43].

In sum, the results of this study indicate that this algal extract may be useful in the control of carbohydrate digestion and absorption. This effect is particularly evident in a mouse model of NASH, obtained by the administration of a high-fat diet. Since NAFLD and NASH may precede the development of T2DM, the use of this natural extract may be exploited therapeutically, to prevent or delay this transition.

## Figures and Tables

**Table 1:** Retention time of methyl esters of fatty acids obtained by means of GC-MS analysis.

Retention Time (min)	Methyl Ester of Fatty Acid
13.75	Methyl myristate
15.88	Methyl hexadecanoate
16.15	Methyl palmitate
18.58	Methyl linoleate
18.68	Methyl oleate
19.09	Methyl stearate
21.22	Methyl arachidonate

**Table 2:** IC_50_ values for α-amylase and α-glucosidase inhibition activity of algal extract and acarbose, used as positive controls.

IC50 Value (μg/mL)		
	α-Amylase	α-Glucosidase
Algal extract	1.49 ± 0.32	0.604 ± 0.004
Acarbose	130.2 ± 2.5	207.2 ± 5.3

Data are presented as means ± SD (*n* = 6). Results are obtained from three independent experiments, performed in duplicate.

## Supplementary Materials

The following are available online at www.mdpi.com/1660-3397/15/2/41/s1, Figure S1: ^1^H-NMR spectrum of the algal extract in deuterated methanol, Figure S2: ^1^H-NMR spectrum of the algal extract in deuterated water, Figure S3: HPLC with gel permeation, Figure S4: GC-MS chromatogram for the identification of methylated fatty acids.
