# Supplementary material for: The Phytocomplex from Fucus vesiculosus and Ascophyllum nodosum Controls Postprandial Plasma Glucose Levels: An In Vitro and In Vivo Study in a Mouse Model of NASH
Source: Mar Drugs. 2017 Feb 15;15(2):41. doi: 10.3390/md15020041 (PMC5334621; doi:10.3390/md15020041)
Supplement: Supplementary file 1 [file marinedrugs-15-00041-s001.pdf]

Supplementary Materials: Phytocomplex from *Fucus vesiculosus* and *Ascophyllum nodosum* Controls Postprandial Plasma Glucose Levels: An In Vitro and In Vivo Study in a Mouse Model of NASH

Daniela Gabbia, Stefano Dall'Acqua, Iole-Maria Di Gangi, Sara Bogialli, Valentina Caputi, Laura Albertoni, Ilaria Marsilio, Nicola Paccagnella, Maria Carrara, Maria Cecilia Giron and Sara De Martin

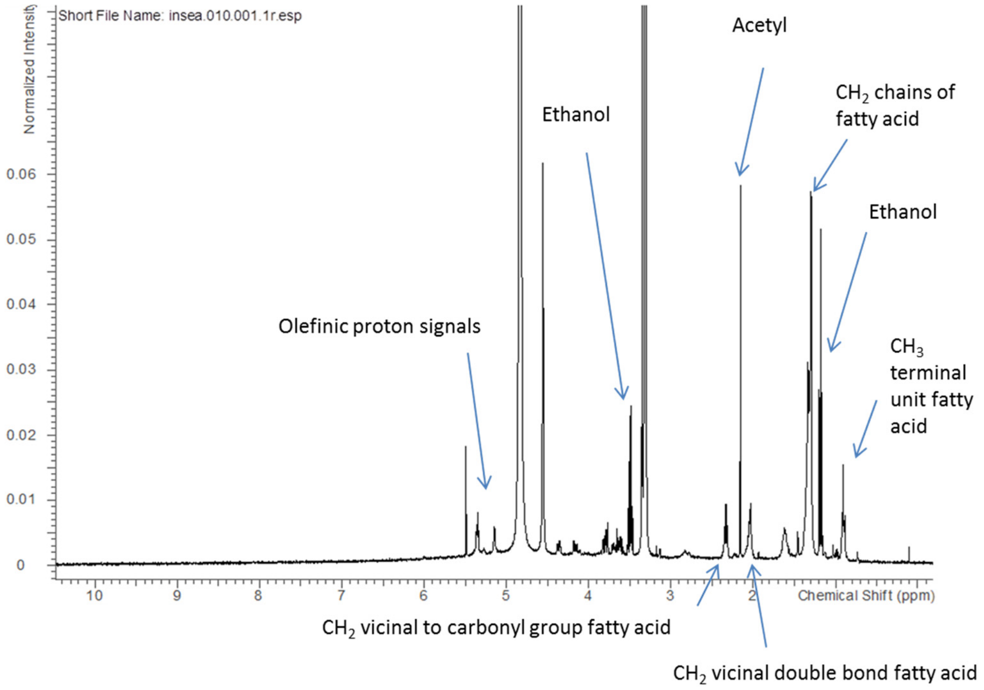

Figure S1. ^{1}H-NMR spectrum of the algal extract in deuterated methanol.

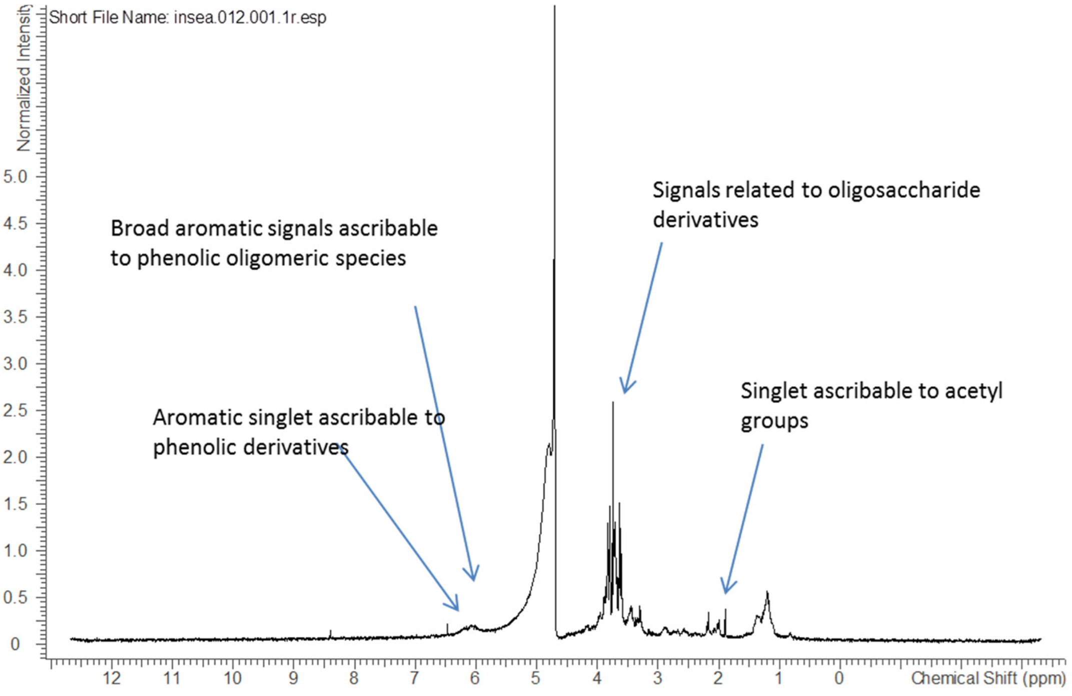

Figure S2. ^{1}H-NMR spectrum of the algal extract in deuterated water.

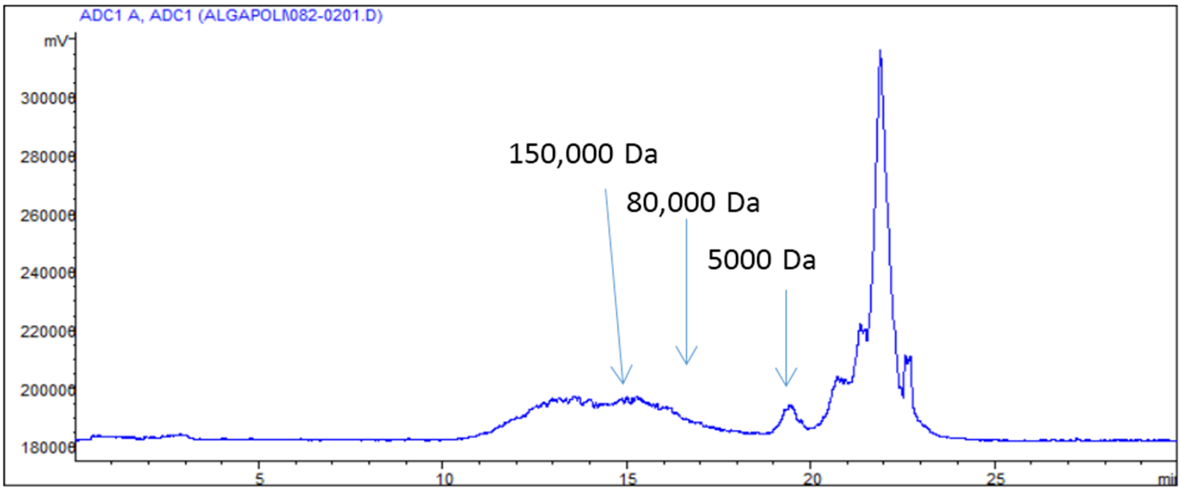

Figure S3. HPLC with gel permeation.

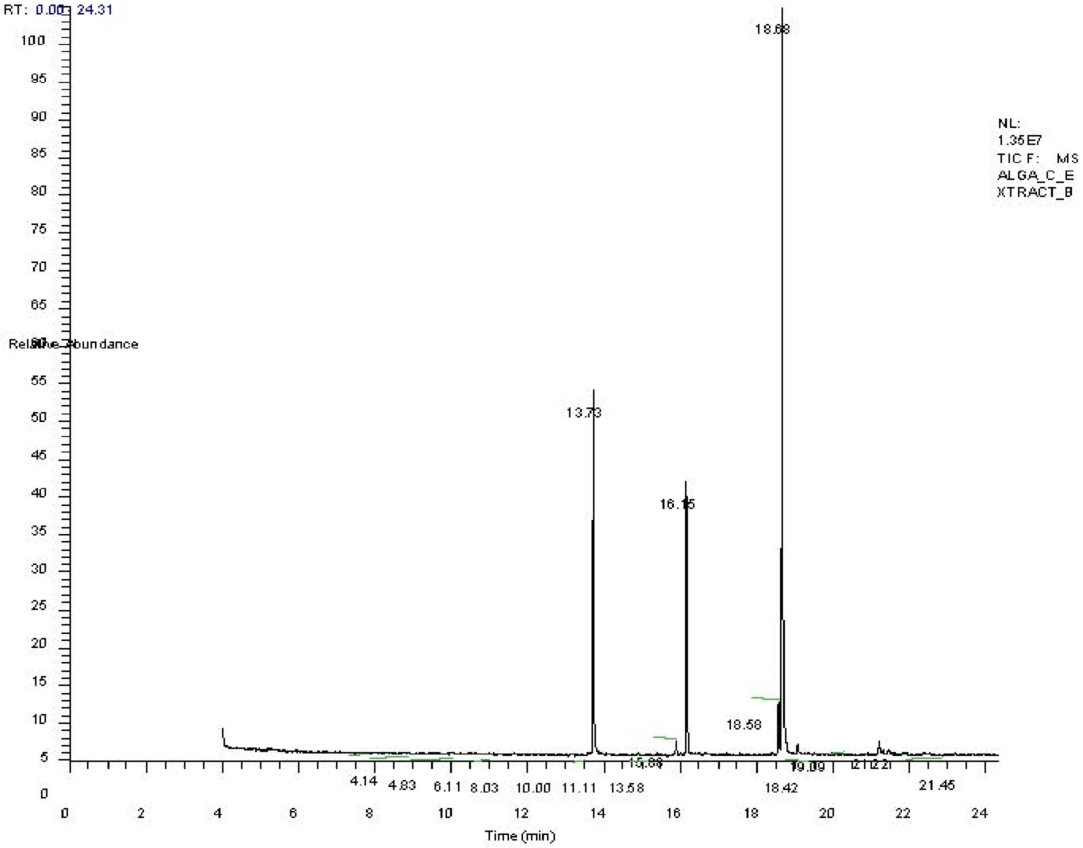

Figure S4. GC-MS chromatogram to identification of methylated fatty acids.
